# Strategies for implementation of a transmural fall-prevention care pathway for older adults with fall-related injuries at the emergency department

**DOI:** 10.1186/s12873-024-01085-9

**Published:** 2024-10-11

**Authors:** W. M. Charmant, B. A. M. Snoeker, H. P. J. van Hout, E. Geleijn, N. van der Velde, C. Veenhof, P. W. B. Nanayakkara

**Affiliations:** 1 Section General Internal Medicine, Department of Internal Medicine, Amsterdam UMC Location VUmc, Amsterdam, The Netherlands; 2 Amsterdam Public Health Research Institute, Amsterdam, The Netherlands; 3 Departments of General Practice & Medicine for Older Persons, Amsterdam UMC Location VUmc, Amsterdam, The Netherlands; 4 Department of Rehabilitation Medicine, Amsterdam UMC Location VUmc, Amsterdam, The Netherlands; 5 Department of Internal Medicine, Section of Geriatric Medicine, Amsterdam UMC, University of Amsterdam, Amsterdam, The Netherlands; 6 Department of Rehabilitation, Physical Therapy Science and Sport, Brain Center, University Medical Center Utrecht, Utrecht University, Utrecht, The Netherlands; 7 Research Centre for Healthy and Sustainable Living, Innovation of Movement Care Research Group, HU University of Applied Sciences Utrecht, Utrecht, The Netherlands

**Keywords:** Implementation, Older adults, Fall prevention, Transmural care, Emergency department

## Abstract

**Background:**

Although indicated, referrals for multifactorial fall risk assessments in older adults with fall related injuries presenting at the emergency department (ED) are not standard. The implementation of a transmural fall-prevention care pathway (TFCP) could bridge this gap by guiding patients to multifactorial fall risk assessments and personalised multidomain interventions in primary care. This study aims to develop and evaluate implementation strategies for a TFCP.

**Methods:**

In this mixed-methods implementation study, strategies were developed using the Consolidated Framework for Implementation Research Expert Recommendations for Implementing Change Matching Tool. These were evaluated with patients, involved healthcare professionals, and other stakeholders using the Reach, Adoption, Implementation, and Maintenance of the RE-AIM framework in two cycles. Patients of the TFCP consisted of frail community dwelling individuals aged 65 and over presenting at the ED with fall related injuries.

**Results:**

During the first implementation phase, strategies were focussed on assessing readiness, adaptability, local champions, incentives and education for all involved healthcare professions in the TFCP. Only 34.4% of eligible patients were informed of the TFCP at the ED, 30.6% agreed to a fall risk assessment and 8.3% patients received the fall risk assessment. In the second phase, this improved to 67.1%, 64.6%, and 35.4%, respectively. Strategies in this phase focussed on adaptability, obtaining sustainable financial resources, local champions, assessing readiness, and education. The implementation was facilitated by strategies related to awareness, champion recruitment, educational meetings, adaptability of TFCP elements and evaluations of facilitators and barriers.

**Conclusion:**

The study outlined strategies for implementing TFCPs in EDs. Strategies included increasing awareness, utilising local champions, educational initiatives, adaptability of the TFCP, and continuous monitoring of facilitators and barriers. These insights can serve as a blueprint for enhancing fall prevention efforts for older adults in emergency department settings.

**Supplementary Information:**

The online version contains supplementary material available at 10.1186/s12873-024-01085-9.

## Background

The increase in patients aged 65 years and older presenting with fall-related injuries at the emergency department (ED) is a major public health concern [1]. According to the World Guidelines for Falls Prevention and Management, older adults visiting the ED with fall-related injuries are at high risk for recurrent falls [2–5]. Up to 23.3% has a recurrent fall within a year and 36.5% has an ED revisit or death within a year [6, 7]. Recurrent falls increase dependency, poor quality of life, and mortality risk [8–10]. An integrated transmural care approach is necessary to ensure that high risk patients receive a multifactorial fall risk assessment following an ED visit to implement personalised multidomain interventions, as recommended by the guidelines [2, 5, 11]. Despite being responsible for fall preventive actions according to the national guideline, as in most countries, currently it is not standard practice for the ED to refer patients for fall risk assessment and accompanying interventions [2, 12–14].

Previous studies have demonstrated that the ED is a highly effective setting for identifying patients at risk of falling, and successfully referring them onwards to the indicated multifactorial fall risk assessment [7, 15]. Patients are more likely to agree to preventive actions during their ED visit as they are confronted with the consequences of their high fall risk [15].

A transmural fall-prevention care pathway (TFCP) guides patients from the ED to primary care fall prevention interventions. The TFCP includes a transmural connection pathway, a multifactorial fall risk assessment conducted by a primary care healthcare professional, shared decision making moment, a report to the patient’s general practitioner (GP), and accompanying personalised multidomain intervention.

To implement a TFCP, appropriate implementation strategies need to be developed. Currently, it is unknown which strategies would be most successful for implementing a TFCP [16]. A previous study has identified facilitators and barriers to consider when forming the implementation strategies [17]. The results showed that three general themes should be taken into account: communication, organisation and execution, and personal factors.

The aim of this study was to develop and evaluate strategies for the implementation of a TFCP for older adults with fall related injuries at the ED.

## Methods

### Research design

**Fig. 1:**
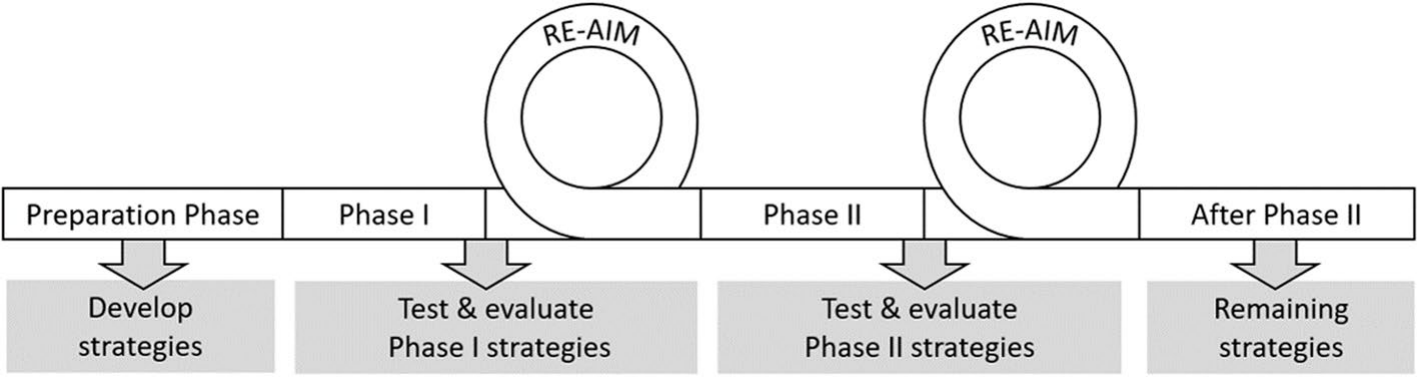
The phases during the implementation study

An action implementation study using mixed-methods with participative elements was conducted to identify strategies for implementing a TFCP from the ED to primary care for older adults. The study, was divided into three phases, the preparation phase, phase I, and phase II (Fig. 1). During the preparation phase, implementation strategies were developed using the Consolidated Framework for Implementation Research Expert Recommendations for Implementing Change (CFIR ERIC) Tool which was filled out based on, known facilitators and barriers [17, 18]. The strategies were specified using principles outlined by *Proctor et al.* (Additional file 1) [19–21]. These strategies were discussed with the consortium of stakeholders including patient organisations, healthcare professionals cooperations, insurance companies, and local governments.

Phase I started in May 2022 and was evaluated with the RE-AIM framework in November 2022 [22]. New strategies were developed during December 2022, which was considered a transition month. Phase II commenced in January 2023 and was evaluated in May 2023.

This study was approved by the Medical Ethical Committee of VU University Medical Centre (METC VUmc 2021.0451). Transparent and accurate reporting was ensured by following the Standards for Reporting Implementation Studies (StaRI) and the Canada Communicable Disease Report checklist for implementation science papers [23, 24]. Non-identifiable patient data from Amsterdam UMC were used in accordance with privacy regulations as part of Amsterdam UMC’s policy for medical scientific research. Written informed consent was obtained from all participants in the evaluations.

### Transmural fall-prevention care pathway

**Fig. 2:**
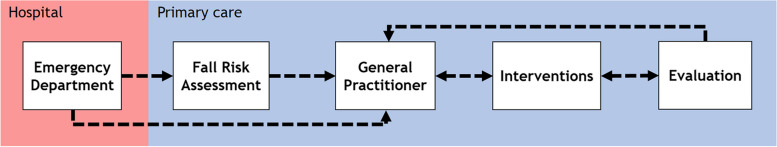
The transmural fall-prevention care pathway

In the Netherlands, patients are referred to the ED by their GP, brought in by ambulances after an emergency call or they are self-referrals. Self-referrals account for approximately 30% of all ED visits [25]. The process of the TFCP was refined through multiple iterations during the implementation. In the TFCP, patients with fall related injuries at the ED were recognised and informed about the TFCP by ED physicians or nurses. If the patient consented, their contact details were shared with primary care physiotherapists and occupational therapists (PTs) from the TFCP’s network. These PTs were members of the local fall prevention network. The ED physician informed the GP of the referral to the TFCP. The PTs then contacted the patient to schedule a multifactorial fall risk assessment at the patient’s home. The multifactorial fall risk assessment was performed with the InterRAI which screened the falls risk domains as indicated by the world falls guidelines [5, 15]. A personalised plan of multidomain falls preventive interventions was constructed based on the results and shared decision making. The plan could for example constitute of a fall preventive exercise programme and an eye examination at the optician. The PT then reported the plan back to the GP or practice nurse (PN). Two to three months after the multifactorial fall risk assessment, the PT evaluated the patient’s progress with the plan of multidomain interventions (Fig. 2).

### Participants and setting

The TFCP was implemented at the ED of Amsterdam University Medical Centre (UMC), location VUmc, in May 2022. Amsterdam UMC was a collective of two teaching hospitals, location AMC and location VUmc, each with an ED. The ED physicians and nurses mainly worked at one location. The ED physicians and nurses who participated in the study worked at the ED of Amsterdam UMC, location VUmc. The PTs in the study were trained in multifactorial fall risk assessment. The PTs, GPs and PNs all worked in the region of Amsterdam South or Amstelveen.

Eligible patients were 65 years or older and visited the ED due to a fall. The TFCP was specifically for patients who experienced preventable falls due to internal factors, such as tripping, loss of balance, or orthostatic hypotension. Falls resulting from external causes, including traffic accidents, epileptic seizures, or cardiac events, were excluded. In addition, patients had to reside in the suburbs Amsterdam South or Amstelveen and have a Clinical Frailty Scale (CFS) ranging from four to six, meaning very mild to moderate frailty [26]. The CFS was determined by ED physicians. Based on the results of the pilot study, a score lower than four was deemed “too fit” and above six “too frail” [15]. Patients were ineligible if they required surgery or an anticipated long hospital admission of more than 48 h.

### Data collection

The study evaluated the implementation strategies using the RE-AIM framework [22]. This framework monitors the Reach, Effectiveness, Adoption, Implementation, and Maintenance of the TFCP. Quantitative and qualitative measures were used to assess these outcomes. The researcher kept a logbook of interesting events or conversations during the implementation of the TFCP [27].

Reach refers to the absolute number, proportion, and representativeness of individuals who are willing to participate in a given initiative, intervention or program [22]. The progression of eligible patients throughout the TFCP was tracked by monitoring the ED’s electronic patient records and the number of fall risk assessments by PTs. This was used to provide the Reach in four stages throughout the TFCP. The Informing Reach pertained to the number of patients who were informed about the TFCP at the ED. The Agreement Reach concerned the number of patients who consented to commence the TFCP after being informed. The Contact Reach encompassed the number of individuals who had contact with a PT following their agreement. The Assessment Reach concerned the number of patients who had a fall risk assessment following their contact with the PT. Demographic data including age, gender, and city were collected. Data from the electronic patient records were only collected if no research objections were recorded in the patient’s record in accordance with Amsterdam UMC’s policy.

Effectiveness is the impact of an intervention on important outcomes including potential negative effects, quality of life, and economic outcomes [22]. The effectiveness was not measured in this study as the TFCP guided patients to multifactorial fall risk assessments and multidomain interventions with a proven efficacy [28, 29].

Adoption refers to the absolute number, proportion, and representatives of a setting and intervention agents who are willing to initiate a program [22]. The Informing Reach was used as quantitative measure for the Adoption of the ED staff. Interviews were conducted with representative samples of involved healthcare professionals before and after each phase. In these interviews, we discussed the healthcare professionals’ experiences post-Phase I and II, including ED staff familiarity and reasons for PT dropouts.

Implementation refers to the intervention agents’ fidelity to the various elements of an intervention’s protocol, including consistency of delivery as intended and the time required [22]. It also includes adaptations made and the costs in terms of money and time of implementation. These elements were discussed during the interviews with healthcare professionals and patients after each phase. The percentage of ‘perfect’ deliveries, defined as the proportion of all eligible patients who received a fall risk assessment was used as quantitative measure.

Maintenance is the extent to which a behaviour is sustained six months or more after treatment, and a program becomes institutionalised or part of the routine organisation practices and policies [22]. This includes the reasons for maintenance, discontinuance, or adaptation, which was discussed in the interviews with representatives of the involved healthcare professionals. The Informing Reach in the six months following Phase II was used to evaluated the extent to which the TFCP became part of routine organisation practices.

### Data analysis

All quantitative analyses were performed using IBM SPSS Statistics for Windows, version 28 (IBM Corp., Armonk, N.Y., USA). The Informing Reach was calculated by determining the proportion of informed patients at the ED out of all eligible patients. Independent samples t-tests and chi-square tests were used to assess age and gender differences between the informed and uninformed patients at the ED. In the Agreement Reach the number of patients who agreed to start the TFCP were divided by those who were informed of the TFCP. The Contact Reach assessed the proportion of patients who got into contact with a PT out of the patients who agreed to start the TFCP. Finally, the Assessment Reach measured the proportion of patients who received a fall risk assessment out of the patients who got into contact with the PT. The notes from interviews with healthcare professionals and patients were analysed to answer qualitative outcomes of the RE-AIM framework. Based on the quantitative and qualitative results, the research team assessed the perceived contribution of the employed strategies for each setting. The authors categorised the strategies, by absence of an established classification in literature, as having contributed, contributed but required improvement or limited contribution to the implementation. In post-hoc analysis, logistic regression stratified for implementation phase was used to assess the impact of weekdays and weekend on the Informing Reach (Additional file 2).

## Results

### Preparation phase

The research team selected and specified the top five of recommended strategies from the CFIR ERIC Tool (Table 1). Local champions were recruited for the ED, PTs, and GP practices. To address known facilitators and barriers, the research team developed materials to promote and facilitate the TFCP among healthcare professionals. These included inclusion criteria pocket cards for ED nurses and physicians, SmartPhrases or Dotphrases for ED physicians to inform GPs, a website for GPs, newsletters for GPs and a template for the PT’s fall risk assessment report. Additionally, newsletter posters in the restrooms of the ED staff and email newsletters for GPs distributed through the local GP cooperation were created to increase awareness. To increase patient participation, we added a pamphlet for patients and an advice letter from a physician to the materials. Additionally, we recommended the GP to contact the patient after ED discharge and after receiving the PT’s report. An incentive structure was created for ED staff. Separate educational meetings were organised for ED nurses, ED physicians and PTs.

**Table 1:** Specified implementation strategies with targets and justification

Name of the strategy (CFIR)	Action	Targets	Justification
**Phase I**			
Identify and prepare champions	Local champions were identified for each involved healthcare profession.	ED, PTs, GPs	Local champions have a positive impact on clinician behaviour change and help promote awareness [30].
Assess for readiness and identify barriers and facilitators	Use results of the pilot study to improve readiness, facilitators and counter barriers.	Patients, ED, PTs, GPs	Reducing the number of barriers improves the chances of successful adoption [31].
	Evaluate during Phase I which facilitators and barriers for the implementation or TFCP were encountered.	Patients, ED, PTs,	
	Evaluate after Phase I which facilitators and barriers for the implementation or TFCP were encountered.	Patients, ED, PTs, GPs	
Promote adaptability	Adapt processes or materials within the TFCP to the needs from participants or healthcare professionals.	Patients, ED, PTs, GPs	Adoption is a critical aspect to improve the appropriateness or contextual fit of an innovation [32].
Alter incentive/allowance structures	Set incentive team targets for ED physicians and nurses, when the targets are achieved the department receives a small reward.	ED	Rewards can be used as motivational techniques [33].
	Set an individual incentive award, the ED physician or nurse with the most informed patients receives a reward.	ED	
	Provide financial resource for the PTs as regular care does not provide this.	PTs	Innovations need to have adequate financial resources [34, 35]
Conduct educational meetings	Attend the shift handover of ED physicians to educate them about their role in the TFCP.	ED physicians	Educational interventions can improve beliefs and attitudes about evidence based practices in acute care [36].
	Create material to increase awareness of the TFCP.	ED, GP	
	Use the clinical lessons for ED nurses to educate them about their role in the TFCP.	ED nurses	
	Provide the PTs with an e-learning on the fall risk assessments.	PTs	PTs may have a knowledge gap when it comes to using fall risk assessments [37]. Providing education can help improve their understanding [35].
	Conduct meetings to educate the PTs on the TFCP and the fall risk assessment.	PTs	
	Conduct two Q&A’s for PTs with questions on the TFCP or fall risk assessments.	PTs	
**Phase II**			
Promote adaptability	Construct feasible transmural communication.	ED	During Phase I, only a small percentage of the patients contacted the PTs. If contact details are provided, PTs may directly contact the patients.
	Construct reminder for TFCP in the electronic patient file.	ED	Although the ED personnel were familiar with the TFCP, they did not consider it at the appropriate time, resulting in a low reach percentage. Reminders in the electronic patient file can be a trigger.
	Construct a shorter fall risk assessment.	PTs	A shorter fall risk assessment will leave more time for PTs to discuss the plan of multidomain interventions within the same visit.
Identify and prepare champions	Increase the number of local champions at the ED.	ED	Local champions have a positive impact on clinician behaviour change and help promote awareness [30].
Access new funding	Find sustainable financial resources for the PTs to conduct fall risk assessments.	PTs	Limited and fragmented funding is a critical barrier to implementation [34].
Assess for readiness and identify facilitators and barriers	Evaluate during Phase II which facilitators and barriers for the implementation or TFCP were encountered.	ED, PTs,	Reducing the number of barriers improves the chances of successful adoption [31].
	Evaluate after Phase II which facilitators and barriers for the implementation or TFCP were encountered.	Patients, ED, PTs, GPs	
	Implement a reminding system for missed patients.	ED	ED physicians recommended sending a reminder by mail when a patient is missed to increase awareness in similar future cases.
Conduct educational meetings	Attend the shift handover of ED physicians to educate about their role in the TFCP.	ED physicians	Educational interventions can improve beliefs and attitudes about evidence based practices in acute care [36].
	Use the clinical lessons for ED nurses to educate them about their role in the TFCP.	ED nurses	
	Create material to increase awareness of the TFCP.	ED	
	Implement the TFCP in the information provided to new ED physicians and nurses.	ED physicians and nurses	New employees are informed of the TFCP’s existence during their introduction, ensuring they are, at least somewhat, aware of it.
	Provide the new PTs with an e-learning on the fall risk assessments.	New PTs	PTs may have a knowledge gap when it comes to using fall risk assessments [37]. Providing education can help improve their understanding [35].
	Conduct meetings to educate the new PTs on the TFCP and the fall risk assessment.	New PTs	
	Conduct a meeting with the “old” PTs to learn from each other’s experiences.	“Old” PTs	
**After Phase II**			
Access new funding	Provide reimbursement for TFCP’s fall risk assessments.	PTs	Reimbursement is necessary to cover the costs of care; excluding EBPs from fee-for-service lists/formularies disincentivises their use [34].
Promote adaptability	Construct feasible transmural communication.	ED	During Phase II, the transmural referral letters did not work optimal. Although it did show promising results, a sustainable alternative must be developed.
	Construct reminder for TFCP in the electronic patient file.	ED	Construction of the reminder only focussed on ED nurses and was often ignored.
	Construct a SmartPhrase that automatically appear in the discharge letter.	ED	In Phase I, an ED physician indicated that the SmartPhrase could be easily forgotten in the future. An automatic SmartPhrase can’t be forgotten.

*ED* Emergency department, *PT* Physiotherapist or occupational therapist, *GP* General practitioner, *TFCP* Transmural fall-prevention care pathway, *CFIR* Consolidated framework for implementation research, *EHR* Electronic health record

### Phase I

#### Reach – phase I

**Fig. 3:**
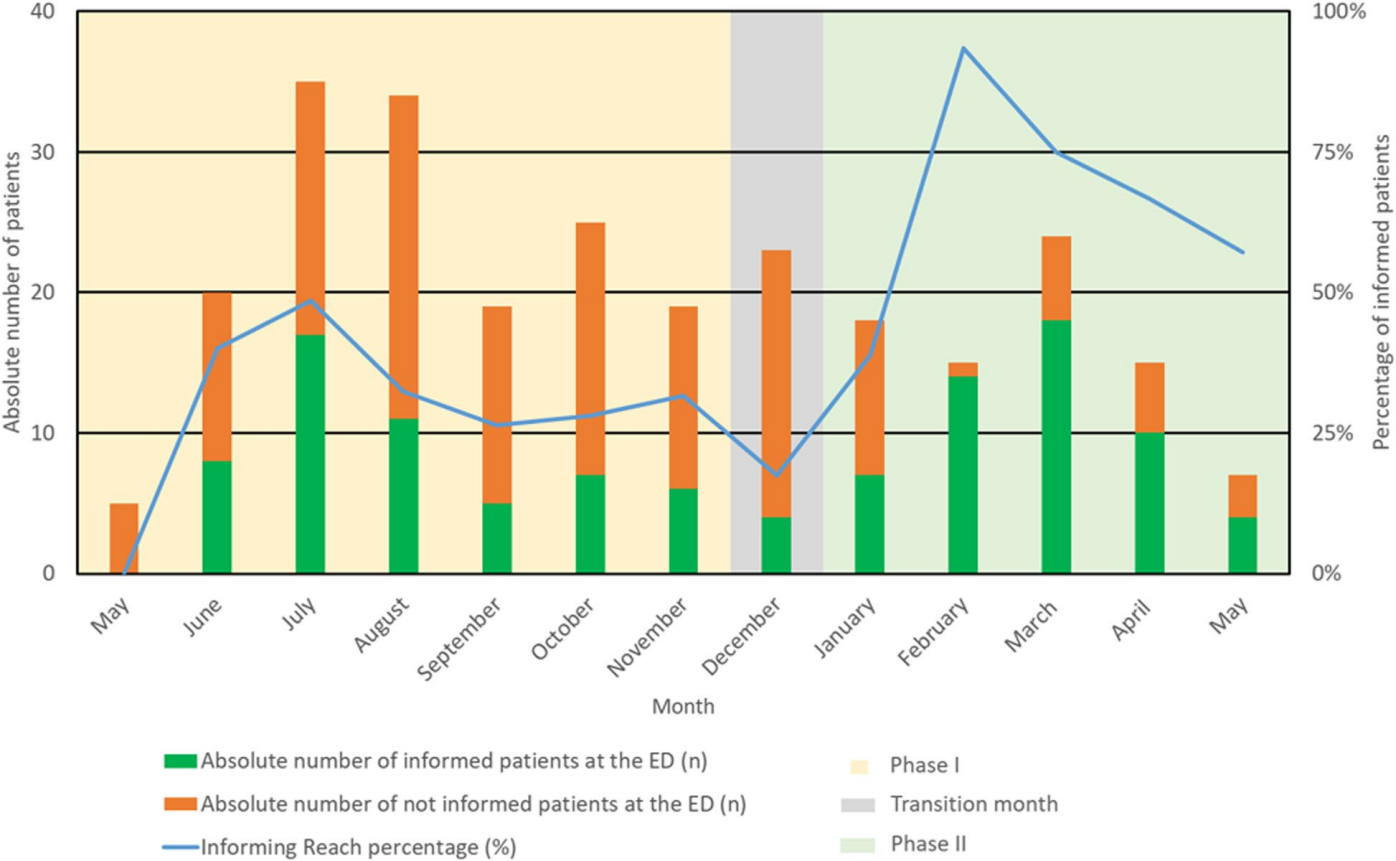
Informing reach during Phase I and Phase II, percentages and absolute numbers. ED = emergency department

A total of 157 eligible TFCP patients arrived at the ED during Phase I, of whom 54 (34.4%, ranging monthly between 0.0% and 48.6%) were informed about the TFCP (Fig. 3). No differences in age and gender were observed between informed and uninformed patients (Table 2). In 48 (88.9%) of the 54 instances patients were informed, they agreed to start the TFCP. Of these patients, 13 (27.1%) contacted the PT to schedule a fall risk assessment appointment. The Informing Reach increased during the first few months as researchers invested more in clinical lessons for ED nurses, explanations during the ED physicians’ shift handovers, and creating awareness. There was a significant difference in Informing Reach between weekdays and weekends during Phase I (*p* < 0.05) (Additional file 2).

**Table 2:** Age and gender of eligible patients (*N* = 157) in phase I

	Informed at ED (*N* = 54)	Not informed at ED (*N* = 103)
Age, years, mean (SD)^†^	82.4 (SD 7.1)	81.4 (SD 7.7)
Gender, female, N (%)^†^	37 (68.5%)	72 (69.9%)

*ED* emergency department, *SD* standard deviation

^†^no significant difference (*p* > 0.05)

#### Adoption – phase I

The overall opinion of the ED staff, PTs, and GPs about the TFCP was positive. In the interviews with ED physicians and nurses, it appeared that they were familiar with the TFCP. However, it remained challenging to apply it at the appropriate time as evidenced by the low Informing Reach. The PT group had only two drop outs, which were unrelated to the TFCP (moving abroad and surgery). GPs and PNs affiliated with the local GP cooperation were familiar with the TFCP. In the interview with PTs, they indicated that GPs, who were not associated with the local GP cooperation, responded surprised when a PT reported the results of the fall risk assessment. According to patient reports, it was uncommon for GPs to contact them about the TFCP after discharge from the ED or after the fall risk assessment by the PT.

#### Implementation – phase I

The percentage of ‘perfect’ TFCP deliveries was 8.3%, as only 13 patients received a multifactorial fall risk assessment. During the interviews, ED staff reported that the TFCP took only a few minutes. ED barriers included not thinking about the TFCP at the right time and low referral rates to PTs. PTs had problems with the duration of the multifactorial fall risk assessment, which took 1 to 1.5 h, making it difficult to discuss a plan of multidomain interventions with the patient. Moreover, PTs experienced the conclusions of the assessment to be too general and lacking recommendations tailored to the patient’s situation. Additionally, scheduling appointments with GPs or PNs was challenging for PTs. The PNs stated that they could be contacted by the PTs via a secure messenger for healthcare professionals.

In accordance with the ‘Promote adaptability’ strategy, several adjustments were made during Phase I (Table 1).

1. A checklist and posters were added for ED physicians and nurses.
2. The inclusion criteria were altered:
  - The patient’s GP didn’t have to be associated with the local GP cooperation as this was too time extensive to check for ED physicians and nurses.
  - The geographic inclusion area was expanded due to a higher number of eligible patients from the neighbouring region Amstelveen than anticipated.
  - Patients could be included if a short admission was expected, decided by ED physicians based on clinical experience.
3. The study’s information folder and physician’s recommendation letter were removed. In conversations with patients, it became apparent that the information of the TFCP, the study and supplements at the ED were overwhelming.
4. Adjustments were made to the SmartPhrase in the Electronic Health Record to clarify the role of the GPs in the TFCP.
5. To increase awareness, the researcher attended ED shift handovers and hospital bed allocation meetings.
6. During the day start meeting, ED champions briefly referred to the TFCP.

#### Maintenance – phase I

ED physicians indicated during the interviews that manually inserting the SmartPhrase should preferably be discontinued in the future. In Phase I, the SmartPhrase was used in 75% of patients who agreed to start the TFCP. The aim should be to make the TFCP comparable to other common injury protocols. Given the high ED staff turnover, the focus of strategies should be on core ED team members. The PTs main Maintenance concern related to the financial compensation for fall risk assessments, with an emphasis on profitability as not all patients require a PT’s intervention.

#### Evaluation of strategies in phase I

The success of educational meetings and awareness creating strategies was confirmed by ED nurses and physicians (Table 3). However, due to high staff turnover, new colleagues had to be informed by peers. The presence of the researcher at the ED, input from local champions, and restroom newsletters were particularly helpful.

*“Every time I see you [the researcher]*,* I think about the TFCP*,* but that is intended right?” – ED physician 1*.

The ED physicians found pocket cards and posters helpful. The rewards only had short-term impact on awareness. The ED nurses and physicians appreciated the adaptability of the TFCP and the inclusion process.

*“I am happy to see all the steps you have taken to make it as easy as possible for us.” – ED physician 2*.

Local champions were not deemed as necessary among PTs compared to the ED. The educational meetings for PTs were useful, with both e-learning and live meeting being deemed most helpful. However, PTs indicated that the fall risk assessment took too long. They did appreciate the e-learning and live meeting. The online meeting seemed redundant, as it covered similar content to the e-learning. The Q&A’s sessions could have been replaced with email correspondence.

Some of the PNs who received reports from the PTs indicated that they were familiar with the TFCP through the newsletters of the local GP organisation. The PNs were satisfied with the SmartPhrase used by the ED in the discharge letter.

**Table 3:** Evaluation of the implementation strategies for phase I and Phase II, and their contribution to implementation as perceived by the research team

Name of the strategy (CFIR)	Action	Perceived contribution by the research team			
**Phase I**		Patients	ED	PT	GP
Identify and prepare champions	Local champions were identified for each involved healthcare profession.	*NA*	+	+/-	+/-
Assess for readiness and identify barriers and facilitators	Use results of the pilot study to improve readiness, facilitators and counter barriers.	++	++	++	++
	Evaluate during Phase I which facilitators and barriers for the implementation or TFCP were encountered.	++	++	++	*NA*
	Evaluate after Phase I which facilitators and barriers for the implementation or TFCP were encountered.	++	++	++	++
Promote adaptability	Adapt processes or materials within the TFCP to the needs from participants or healthcare professionals.	++	++	++	+/-
Alter incentive/allowance structures	Set incentive team targets for ED physicians and nurses, when the targets are achieved the department receives a small reward.	*NA*	+/-	*NA*	*NA*
	Set an individual incentive award, the ED physician or nurse with the most informed patients receives a reward.	*NA*	+/-	*NA*	*NA*
	Provide financial resource for the PTs as regular care does not provide this.	*NA*	*NA*	+	*NA*
Conduct educational meetings	Attend the shift handover of ED physicians to educate them about their role in the TFCP.	*NA*	++	*NA*	*NA*
	Create material to increase awareness of the TFCP.	*NA*	*++*	*NA*	*++*
	Use the clinical lessons for ED nurses to educate them about their role in the TFCP.	*NA*	++	*NA*	*NA*
	Provide the PTs with an e-learning on the fall risk assessments.	*NA*	*NA*	++	*NA*
	Conduct meetings to educate the PTs on the TFCP and the fall risk assessment.	*NA*	*NA*	+	*NA*
	Conduct two Q&A’s for PTs with questions on the TFCP or fall risk assessments.	*NA*	*NA*	+/-	*NA*
**Phase II**					
Promote adaptability	Construct feasible transmural communication.	*NA*	+	*NA*	*NA*
	Construct reminder for TFCP in the electronic patient file.	*NA*	+/-	*NA*	*NA*
	Construct a shorter fall risk assessment.	*NA*	*NA*	++	*NA*
Identify and prepare champions	Increase the number of champions at the ED.	*NA*	++	*NA*	*NA*
Access new funding	Find sustainable financial resources for the PTs to conduct fall risk assessments.	*NA*	*NA*	+	*NA*
Assess for readiness and identify facilitators and barriers	Evaluate during Phase II which facilitators and barriers for the implementation or TFCP were encountered.	++	++	++	++
	Evaluate after Phase II which facilitators and barriers for the implementation or TFCP were encountered.	++	++	++	++
	Implement a reminding system for missed patients.	*NA*	++	*NA*	*NA*
Conduct educational meetings	Attend the shift handover of ED physicians to educate about their role in the TFCP.	*NA*	++	*NA*	*NA*
	Use the clinical lessons for ED nurses to educate them about their role in the TFCP.	*NA*	++	*NA*	*NA*
	Create material to increase awareness of the TFCP.	*NA*	*++*	*NA*	*NA*
	Implement the TFCP in the information provided to new ED physicians and nurses.	*NA*	+/-	*NA*	*NA*
	Provide the new PTs with an e-learning on the fall risk assessments.	*NA*	*NA*	++	*NA*
	Conduct meetings to educate the new PTs on the TFCP and the fall risk assessment.	*NA*	*NA*	++	*NA*
	Conduct a meeting with the “old” PTs to learn from each other’s experiences.	*NA*	*NA*	++	*NA*

*ED* Emergency department, *PT* Therapists, *GP* General dractitioner, *NA* Not applicable

++ = contributed, + = contributed but required improvement, +/- = limited contribution

### Forming implementation strategies for phase II

In preparation of Phase II, we focused our strategies on three main issues (Table 1). Firstly, to increase the Informing Reach, we created a reminder question for ED nurses in the EHR, increased the number of local champions in the ED and initiated email reminders to ED physicians after missed TFCP patients. Secondly, to increase the Contact Reach, we created an automatically generated transmural referral letter with contact details to the PTs. The letter was received by two key PTs. Using a group chat in a secure messaging platform for healthcare professionals with all participating PTs, the key PTs would indicate the area in which the referred patient resided. The first responding PT would then receive the patient’s contact details in a private message. Thirdly, to reduce the PTs time costs, around 100 items from the InterRAI questionnaire, that were not essential for the multifactorial fall risk assessment like abuse and driving, were removed [5]. Additionally, a live meeting for PTs was organised to exchange knowledge and experiences.

### Phase II

#### Reach – phase II

**Fig. 4:**
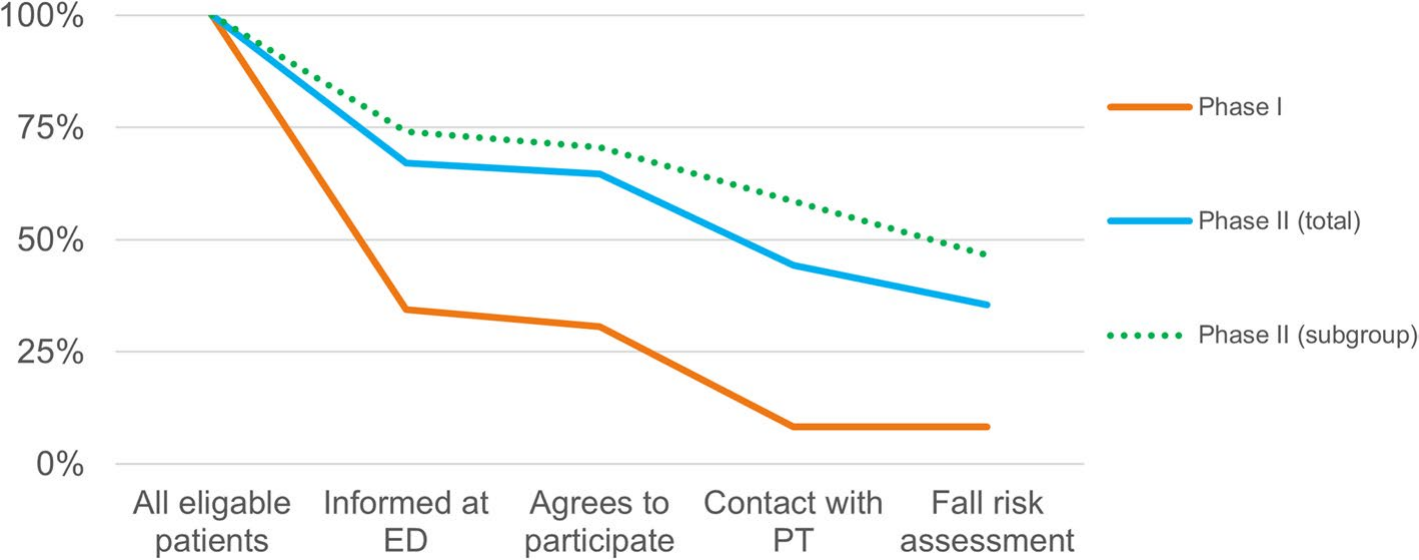
Patient flow in the TFCP during Phase I, Phase II (total) and the Phase II-subgroup after the completion of the email reminder and transmural referral letter strategies. ED = emergency department, PT = physio- or occupational therapist

Out of the 79 eligible TFCP patients, 53 (67.1%) were informed by the ED, with monthly rates ranging from 38.9 to 93.3% (Figs. 3 and 4). Of these, 51 (96.2%) agreed to start the TFCP, 35 (68.6%) got into contact with a PT, and 28 (80.0%) received a multifactorial fall risk assessment. No significant differences in age or gender between informed and uninformed patients were observed (Table 4). Contrary to Phase I, the difference in Informing Reach between weekends and weekdays was no longer significant in Phase II (*p* > 0.05) (Additional file 2).

In-depth analysis showed different Reaches in subgroups after completion of two delayed strategies. The Informing Reach increased to 75% (51/68) after the start of email reminders to ED physicians on January 23rd. The Contact Reach increased to 82.9% (34/41) following the transmural referral letters in the EHR on February 6th. The green dotted line in Fig. 4 illustrates the impact of these strategies on the patients flow after February 6th.

However, changes in acute care infrastructure at Amsterdam UMC in late March reduced the number of eligible patients from 25 to 8 per month, leading to a decrease in Informing Reach. Notably, during the transition month of December 2022, the Informing Reach dropped to 17.4% due to the researcher’s absence.

**Table 4:** Age and gender of eligible patients (*N* = 79) during phase II

	Informed at ED (*N* = 53)	Not informed at ED (*N* = 26)
Age, years, mean (SD)^†^	81.0 (SD 6.8)	83.2 (SD 7.8)
Gender, female, N (%)^†^	33 (62.3%)	16 (61.5%)

*ED* emergency department, *SD* standard deviation

^†^ no significant difference (*p* > 0.05)

#### Adoption – phase II

In Phase II the Adoption improved compared to Phase I, with increased awareness among ED staff, reflected in the higher Informing Reach. Only one PT dropped out due to pregnancy leave. The biggest challenge in adoption was observed in the participation of PTs. Some PTs responded promptly to fall risk assessment offers in the group chat while others did not. In the interviews they indicated that for some this was due to busy schedules.

#### Implementation – phase II

In general, the percentage of ‘perfect’ TFCP deliveries increased to 35.4% as 28 patients received multifactorial fall risk assessments in Phase II. This was mainly caused by the completion of the transmural referral letter strategy as subgroup analysis showed an increase to 46.6% (27/58) of ‘perfect’ deliveries (Fig. 4).

Technical issues caused the referral letters to be unavailable to a large portion of the ED staff. To circumvent this issue and still assess the potential impact of the referral letter strategy, the researcher provided the PTs with the contact details if ED physicians recorded patients’ consent in their notes. Consent was provided by 85.4% of patients who were interested in the TFCP. As the note required an additional action by the ED physician which was comparable to sending the referral letter through the EHR, we regarded this as the same action.

The increased demand for multifactorial fall risk assessments resulted in the training of additional PTs. The shorter multifactorial fall risk assessment took PTs between 35 and 50 min to complete. PNs mentioned increasing awareness of the TFCP among GP assistants as they could aid PTs in scheduling appointments with the GP or PN.

#### Maintenance – phase II

During the interviews, the ED physicians were curious how the TFCP would function after study completion, as the researcher’s presence at the ED acted as a reminder. They suggested sharing weekly success stories through the ED newsletter to maintain its relevance. They also discussed a pop-up or reminder in the EHR based on the patient’s age and living area. ED nurses helped to remind physicians about the TFCP or wrote a note in the patient’s EHR. Although the SmartPhrase was manually inserted in 89.1% of the discharge letters of Phase II, automating its addition may be beneficial in the future. In the six months following Phase II, the Informing Reach was 53.6% (ranging from 25.0 to 83.3%), affected by changes in acute care infrastructure. The lower Informing Reaches occurred during months with more shifts by ED doctors primarily based at location AMC, who were less familiar with the TFCP.

#### Evaluation of strategies in phase II

The EHR reminder question had limited contribution, as it was easily overlooked (Table 3). The option for transmural referral letters in the EHR needed improvement as it was only visible to ED supervisors. Sharing contact details with PTs positively impacted the Contact Reach, increasing from 27.1 to 82.9%. The ED physicians reported that the mailing strategy had a positive impact on the implementation, increasing their awareness for future cases, as one ED physician jokingly remarked:

[Jokingly] “*I don’t want to receive another one of those [reminder] emails*.” – ED physician 3.

The new and old ED champions, clinical lessons for ED nurses, and short briefings during shift handovers for ED physicians contributed to the increased awareness according to ED staff. Healthcare professionals valued the assessment of facilitators and barriers as it would lead to further improvements and increased efficiency. During Phase II, sustainable resources for PTs’ fall risk assessments were not secured.

### Post phase II strategies

After Phase II, the remaining strategies focussed on implementing the reminder and transmural referral letter features in the EHR, automating the SmartPhrase, and establishing sustainable financial resources for PTs (Table 1).

## Discussion

The results from this paper, outlining the development of implementation strategies for the implementation of a TFCP for older adults with fall-related injuries at the ED, demonstrated contributions in Reach, Adoption, Implementation, and Maintenance. Strategies focused on increasing awareness, utilising local champions, educational initiatives, adaptability of the TFCP, and conducting evaluations of facilitators and barriers. To facilitate implementation in other hospitals and regions, a blueprint based on the experiences from this study was developed (Additional file 3).

The ED presents a unique setting for implementing a TFCP, as this fast-paced environment traditionally focuses on acute care and immediate medical interventions for urgent health issues, rather than prevention. Older adults visiting the ED with a fall-related injury should be assessed for acute underlying conditions such as pneumonia or myocardial infarction and be guided to fall preventive actions [2, 5]. This shift to proactive medicine requires additional steps from this normally reactive environment. Therefore, the most strategies in this study aimed to change attitudes towards fall prevention. Communication strategies, including face to face contact and regular reminders, were employed to achieve this goal [35]. Raising awareness was facilitated by the researcher’s presence at the ED. The researcher unintentionally became a local champion for the TFCP, as his mere presence influenced behaviour and impacted the Informing Reach, as evidenced by the drop to 17.4% in his absence during December 2022. The physical presence of champions is one of the six key attributes previously described alongside influence, ownership, persuasiveness, grit, and participative leadership style [38]. The strategies effectively integrated the TFCP into the daily routine of ED staff over time, which is illustrated by the improvements in Informing Reach and the disappearance of the difference in Informing Reach between weekdays and weekends during Phase II.

EDs experience a high degree of nursing staff turnover and the physicians are a select group of attending physicians who are accompanied by an ever-changing cycle of medical students, junior physicians, and residents [39]. In line with our findings this problem, amplified by the COVID-19 pandemic, also influenced the implementation of a geriatric screening program at the ED in the United States [40]. The authors discovered that involving local champions from both bedside and leadership staff could have improved implementation. Similar to our study, the authors demonstrated an improvement in Reach after employing a combination of on-shift education and educational email strategies. However, caution in framing the emails is advised as they can be perceived as unhelpful and have a counter effective result [41]. Another study which implemented a fall preventive care pathway at the ED also found that active engagement of involved staff and locally-adapted referral pathway contributed to the implementation [42]. Furthermore, they found that an audit and feedback of performance indicators could contribute. Alerts in the EHR have been suggested as planned improvements to implementation and have shown to contribute to implementation efforts [43–45].

Previous studies have identified seven themes that should be taken into account when implementing a care pathway in the ED. These themes, derived from the Theoretical Domains Framework, include: care pathway tools and standardisation, patient-specific issues, professional issues, team dynamics, strategies for success and sustainability, hospital resources and processes, and quality and process improvement [46]. In our study, all seven identified themes played a role, with particular emphasis on care pathway tools and standardisation and team dynamics. Streamlining the TFCP process was a central focus, including EHR tool integrations. The team dynamics including the local champions and researcher influenced the awareness of the ED staff and the Informing Reach throughout the implementation. The authors stress the importance of considering these factors within the context of the individual, the health team and the hospital organisation when using multifaceted strategies [46, 47]. Addressing the context using adaptability strategies is one of the priorities in implementation science as it influences outcomes like Reach, Adoption and Maintenance [48].

The adaptation of the procedure of contact after ED discharge impacted the Contact Reach. Initially, when patients agreed to start the TFCP in Phase I, only 27.1% got into contact with a PT as they were required to contact the PTs themselves. Patients’ hesitancy to contact PTs may stem from their lack of identification with the TFCP’s target population [17]. After implementing transmural referral letter strategy that provided PTs with patients’ contact details in Phase II, the Contact Reach increased to 82.9%. This change ensured patients were followed up, while still giving them the option to refuse the assessment. The researcher manually sent referral letters due to EHR issues that made the automated system unavailable. Letters were sent only when ED physicians recorded patient consent in the EHR notes, requiring effort similar to the automated system’s two to three clicks. This adjustment, necessary to circumvent the EHR problems, closely aligned with the original protocol’s effort and provided the opportunity to test the referral letter’s potential. A benefit of offering the option of PT-initiated calls is that patients can receive a second explanation of the TFCP. During an ED visit, patients were often emotional or confused, making it difficult to remember information [17]. In 85.4% of cases, patients provided consent to share their contact details, and of those called 80.0% still wanted the fall risk assessment. This highlights the potential of an operational transmural link.

### Strengths and limitations

A limitation of the study was the inability to track patients over time to assess their engagement with fall prevention interventions. This was caused by the confusing effect of having both the study information and the TFCP information at the ED, which led to reduced participation of patients in the TFCP. To address this, we moved the study information to a later stage of the TFCP, the multifactorial fall risk assessments. Patients were allowed to participate in the TFCP without participating in the study. However, the efforts by PTs to acquire informed consent yielded limited success, resulting in a sample too small to determine an accurate reach of fall prevention interventions.

A potential limitation in this study was the absence of an implementation coordinator as monitored strategy [49]. The implementation coordinator oversees implementation across different settings, maintains contact with local champions and stakeholders from various professions, and addresses any difficulties that arise. In this study, the researcher fulfilled these tasks but the contribution of the strategy was not monitored. Importantly, apart from sending referral letters based on ED physicians’ notes, the researcher did not engage in other TFCP tasks, such as informing patients, scheduling PT appointments, or contacting GPs with fall risk assessment results. Therefore, the researcher’s presence should not be viewed as a limitation but as part of the analysed strategies.

Due to changes in the acute care infrastructure at the conclusion of Phase II, the ED underwent significant changes that made it incompatible with the preceding phases. The majority of strategies were concentrated on guiding the patients from the ED to the multifactorial fall risk assessment. During the first two phases this required more attention than anticipated. A Phase III would have allowed for further analysis of the effect of new strategies, more strategies focussed on guiding patients from the multifactorial fall risk assessment to the multidomain interventions, and a greater focus on the Maintenance of the TFCP.

This study provided a comprehensive, structured and chronological analysis of the implementation process of the TFCP, using the RE-AIM framework to evaluate systematically developed strategies across multiple phases. The recommended mixed quantitative and qualitative approaches were used to assess the outcomes [50]. However, as previously mentioned patient follow-up was limited, limiting the Implementation outcome assessment. Ideally, we would have moved through each step of the TFCP with the participants to deepen understanding of issues as they emerged in practice. The Reach and Adoption were adequately monitored throughout the process with the fragmented Reaches providing quantitative insights in the patient flow and interviews providing the qualitative explanations. Maintenance could only be assessment to some degree due to the changes in the acute care infrastructure.

The strategies are replicable as they are defined by the common CFIR definitions and specified by the fundamentals outlined by *Proctor et al.* (Additional file 1) [18, 19]. The involvement of multiple stakeholders, including patient organisations, healthcare professionals cooperations, insurance companies, and governmental bodies, at several moments throughout the implementation improved the generalisability of the results. The results of this study are applicable to EDs and regions with similar patient demographics, staffing structures, and financial and logistical healthcare organisation. Contexts between implementation settings may vary, and therefore the success of strategies may not necessarily translate to all other settings. However, the strategies outlined in this study can be modified to suit local contexts in discussion with regional partners. The strategy of continued monitoring of facilitators and barriers will help to detect contextual differences on which adaptations may need to be made. Organisational support, financial resources, social relations and support, and leadership are common context dimensions that could differ [51].

## Conclusion

This study presents several implementation strategies for a TFCP for older adults with fall-related injuries at the ED through a systematic approach. The strategies demonstrated contributions in implementation outcomes such as Reach, Adoption, Implementation and Maintenance. Key strategies include increasing awareness, utilising local champions, educational initiatives, adaptability of the TFCP, and continued monitoring of facilitators and barriers. The encountered challenges provided valuable insights into the impact of certain strategies. The results of this study can serve as a blueprint for implementing TFCPs in other ED settings, ultimately improving fall prevention efforts for older adults. Further research should focus on implementation strategies in guiding patients from multifactorial fall risk assessments to multidomain interventions.

## Supplementary Information

Supplementary Material 1.

Supplementary Material 2.

Supplementary Material 3.

## Data Availability

The datasets used and/or analysed during the current study are available from the corresponding author on reasonable request.

## Abbreviations

CFIR ERIC: Consolidated Framework for Implementation Research Expert Recommendations for Implementing Change
CFS: Clinical Frailty Scale
ED: Emergency Department
GP: General Practitioner
PN: Practice Nurse
PT: Physio- or occupational therapist
RE-AIM Framework: Reach Effectiveness Adoption Implementation Maintenance Framework
StaRI: Standards for Reporting Implementation Studies
TFCP: Transmural Fall-prevention Care Pathway
