# Supplementary material for: Strategies for implementation of a transmural fall-prevention care pathway for older adults with fall-related injuries at the emergency department
Source: BMC Emerg Med. 2024 Oct 11;24:188. doi: 10.1186/s12873-024-01085-9 (PMC11470610; doi:10.1186/s12873-024-01085-9)
Supplement: Supplementary file 1 — Supplementary Material 1. [file 12873_2024_1085_MOESM1_ESM.docx]

Additional file 2.

| **Name of the strategy (CFIR)** | **CFIR definition of the strategy** | **Actors** | **Action** | **Targets** | **Temporality** | **Dose** | **Implementation outcome affected** | **Justification** |
| --- | --- | --- | --- | --- | --- | --- | --- | --- |
| **Phase I** | | | | | | | | |
| Identify and prepare champions | Identify and prepare individuals who dedicate themselves to supporting, marketing, and driving through an implementation, overcoming indifference or resistance that the intervention may provoke in an organization. | The research team | Local champions were identified for each involved healthcare profession | ED, PTs, GPs | Preparation phase | At least one local champion in each profession. | Reach, Adoption | Local champions have a positive impact on clinician behaviour change and help promote awareness (30). |
| Assess for readiness and identify barriers and facilitators | Assess various aspects of an organization to determine its degree of readiness to implement *and identify* barriers that may impede implementation and strengths that can be *leveraged to facilitate* the implementation effort. | The research team | Use results of the pilot study to improve readiness, facilitators and counter barriers. | Patients, ED, PTs, GPs | Preparation Phase | - | Implementation, Maintenance | Reducing the number of barriers improves the chances of successful adoption (31). |
|  |  | The research team | Evaluate during Phase I which facilitators and barriers for the implementation or TFCP were encountered | Patients, ED, PTs, | During Phase I | Often during the first months | Reach, Adoption, Implementation, Maintenance |  |
|  |  | The research team | Evaluate after Phase I which facilitators and barriers for the implementation or TFCP were encountered | Patients, ED, PTs, GPs | After Phase I | Once with those active in Phase I | Reach, Adoption, Implementation, Maintenance |  |
| Promote adaptability | Identify the ways a clinical innovation can be tailored to meet local needs and clarify which elements of the innovation must be maintained to preserve fidelity | The research team | Adapt processes or materials within the TFCP to the needs from participants or healthcare professionals | Patients, ED, PTs, GPs | During Phase I | As much as necessary | Reach, Adoption, Implementation, Maintenance | Adoption is a critical aspect to improve the appropriateness or contextual fit of an innovation (32). |
| Alter incentive/allowance structures | Work to incentivize the adoption and implementation of the clinical innovation | The research team | Set incentive team targets for ED doctors and nurses, when the targets are achieved the department receives a small reward. | ED | During Phase I | Once | Reach | Rewards can be used as motivational techniques (33). |
|  |  | The research team | Set an individual incentive award, the ED doctor or nurse with the most informed patients receives a reward. | ED | At the end of Phase I | Once | Reach |  |
|  |  | The research team | Provide financial resource for the PTs as regular care does not provide this | PTs | During Phase I | For each fall risk assessment | Reach, Adoption, Implementation. | Innovations need to have adequate financial resources (34, 35) |
| Conduct educational meetings | Hold meetings targeted toward *educating* *multiple* stakeholder groups (i.e. providers, administrator*s*, other organizational stakeholders, community *members*, patient*s*/consumer*s*, famil*ies*) *about the clinical innovation and/or its implementation.* | The research team | Attend the shift handover of ED doctors to educate them about their role in the TFCP | ED doctors | Preparation Phase and during Phase I | 19 times | Reach, Adoption | Educational interventions can improve beliefs and attitudes about evidence based practices in acute care (36). |
|  |  | The research team | Create material to increase awareness of the TFCP | ED, GP | Preparation Phase, Phase I | 9 times | Reach, Adoption |  |
|  |  | The research team | Use the clinical lessons for ED nurses to educate them about their role in the TFCP | ED nurses | Preparation Phase and during Phase I | 5 times | Reach, Adoption |  |
|  |  | The research team | Provide the PTs with an e-learning on the fall risk assessments | PTs | Preparation Phase | 1 time | Implementation | PTs may have a knowledge gap when it comes to using fall risk assessments (37). Providing education can help improve their understanding (35). |
|  |  | The research team | Conduct meetings to educate the PTs on the TFCP and the fall risk assessment | PTs | Preparation Phase and during Phase I | Two sessions | Implementation |  |
|  |  | The research team | Conduct two Q&A’s for PTs with questions on the TFCP or fall risk assessments | PTs | During Phase I | Two sessions | Implementation |  |
| **Phase II** | | | | | | | | |
| Promote adaptability | Identify the ways a clinical innovation can be tailored to meet local needs and clarify which elements of the innovation must be maintained to preserve fidelity | The research team and EHR support | Construct feasible transmural communication | ED | During Phase II | - | Reach | During Phase I, only a small percentage of the patients contacted the PTs. If contact details are provided, PTs may directly contact the patients. |
|  |  | The research team and EHR support | Construct reminder for TFCP in the electronic patient file | ED | During Phase II | - | Reach | Although the ED personnel were familiar with the TFCP, they did not consider it at the appropriate time, resulting in a low reach percentage. Reminders in the electronic patient file can be a trigger. |
|  |  | The research team and fall risk assessment software team | Construct a shorter fall risk assessment | PTs | Phase II | - | Implementation | A shorter fall risk assessment will leave more time for PTs to discuss the plan of multidomain interventions within the same visit. |
| Identify and prepare champions | Identify and prepare individuals who dedicate themselves to supporting, marketing, and driving through an implementation, overcoming indifference or resistance that the intervention may provoke in an organization. | The research team | Increase the number of local champions at the ED | ED | Phase II | At least one additional local ED champion | Reach, Adoption | Local champions have a positive impact on clinician behaviour change and help promote awareness (30). |
| Access new funding | Access new or existing money to facilitate the implementation | The research team and government? | Find sustainable financial resources for the PTs to conduct fall risk assessments | PTs | Phase II | - | Implementation | Limited and fragmented funding is a critical barrier to implementation (38). |
| Assess for readiness and identify facilitators and barriers | Assess various aspects of an organization to determine its degree of readiness to implement *and identify* barriers that may impede implementation and strengths that can be *leveraged to facilitate* the implementation effort. | The research team | Evaluate during Phase II which facilitators and barriers for the implementation or TFCP were encountered | ED, PTs, | During Phase II | Often during the first months | Reach, Adoption, Implementation, Maintenance | Reducing the number of barriers improves the chances of successful adoption (31). |
|  |  | The research team | Evaluate after Phase II which facilitators and barriers for the implementation or TFCP were encountered | Patients, ED, PTs, GPs | After Phase II | Once with those active in Phase II | Reach, Adoption, Implementation, Maintenance |  |
|  |  | The research team | Implement a reminding system for missed patients | ED | During Phase II | During Phase II after all missed patients | Reach, Adoption | ED doctors recommended sending a reminder by mail when a patient is missed to increase awareness in similar future cases. |
| Conduct educational meetings | Hold meetings targeted toward *educating* *multiple* stakeholder groups (i.e. providers, administrator*s*, other organizational stakeholders, community *members*, patient*s*/consumer*s*, famil*ies*) *about the clinical innovation and/or its implementation.* | The research team | Attend the shift handover of ED doctors to educate about their role in the TFCP | ED doctors | During Phase II | 15 times | Reach, Adoption | Educational interventions can improve beliefs and attitudes about evidence based practices in acute care (36). |
|  |  | The research team | Use the clinical lessons for ED nurses to educate them about their role in the TFCP | ED nurses | During Phase II | 6 times | Reach, Adoption |  |
|  |  | The research team | Create material to increase awareness of the TFCP | ED | Phase II | 10 times | Reach, Adoption |  |
|  |  | The research team | Implement the TFCP in the information provided to new ED doctors and nurses. | ED doctors and ED nurses | Phase II | - | Maintenance | New employees are informed of the TFCP's existence during their introduction, ensuring they are, at least somewhat, aware of it. |
|  |  | The research team | Provide the new PTs with an e-learning on the fall risk assessments. | New PTs | Phase II | 1 time | Implementation | PTs may have a knowledge gap when it comes to using fall risk assessments (37). Providing education can help improve their understanding (35). |
|  |  | The research team | Conduct meetings to educate the new PTs on the TFCP and the fall risk assessment. | New PTs | Preparation Phase and during Phase I | Two sessions | Implementation |  |
|  |  | The research team | Conduct a meeting with the “old” PTs to learn from each other’s experiences. | “Old” PTs | Phase II | One session | Implementation |  |
| **After Phase II** | | | | | | | | |
| Access new funding | Access new or existing money to facilitate the implementation | Insurance companies | Provide reimbursement for TFCP’s fall risk assessments. | PTs | After Phase II | - | Implementation | Reimbursement is necessary to cover the costs of care; excluding EBPs from fee-for-service lists/formularies disincentivises their use (38). |
| Promote adaptability | Identify the ways a clinical innovation can be tailored to meet local needs and clarify which elements of the innovation must be maintained to preserve fidelity | The research team and EHR support | Construct feasible transmural communication | ED | After Phase II | - | Reach | During Phase II, the transmural referral letters did not work optimal. Although it did show promising results, a sustainable alternative must be developed. |
|  |  | The research team and EHR support | Construct reminder for TFCP in the electronic patient file | ED | After Phase II | - | Reach | Construction of the reminder only focussed on ED nurses and was often ignored. |
|  |  | The research team and EHR support | Construct a SmartPhrase that automatically appear in the discharge letter. | ED | After Phase II | - | Implementation | In Phase I, an ED doctor indicated that the SmartPhrase could be easily forgotten in the future. An automatic SmartPhrase can’t be forgotten. |
