# Supplementary material for: Strategies for implementation of a transmural fall-prevention care pathway for older adults with fall-related injuries at the emergency department
Source: BMC Emerg Med. 2024 Oct 11;24:188. doi: 10.1186/s12873-024-01085-9 (PMC11470610; doi:10.1186/s12873-024-01085-9)
Supplement: Supplementary file 2 — Supplementary Material 2. [file 12873_2024_1085_MOESM2_ESM.docx]

A logistic regressions was performed to assess the differences in Informing Reach between weekdays and weekends (Table 1 in Additional file 2). A significant association between Informing Reach and whether it was a weekday or weekend was observed, independent of implementation phase (p = 0.028). The Informing Reach was lower in patients who visited during the weekend (32.4% 22/68) than during the weekdays (51% 85/168). The odds to be informed during the weekend were 0.500 times (95% CI 0.269-0.927) the odds to be informed during the weekdays.

Additional file 2 Table 1. Logistic regression analysis.

| **Variable** | **B** | **S.E.** | **Wald** | **df** | **p-value** | **OR** | **95% CI** |
| --- | --- | --- | --- | --- | --- | --- | --- |
| Weekday or weekend | -0.694 | 0.315 | 4.836 | 1 | 0.028 | 0.500 | 0.269 – 0.927 |
| Implementation phase | 1.325 | 0.296 | 20.108 | 1 | < 0.001 | 3.764 | 2.109 – 6.718 |
| Constant | -1.768 | 0.426 | 17.262 | 1 | < 0.001 | 0.171 |  |

B = Regression coefficient, S.E. = Standard Error, df = degrees of freedom, OR = Odds Ratio and 95% CI = 95% Confidence Interval

Further analysis showed that the impact of weekdays and weekends disappeared during Phase II (Table 2 in Additional file 2). During Phase I, the Informing Reach was 40.2% (43/107) during weekdays and 22.0% (11/50) during weekends. During Phase II, this was 68.9% (42/61) during weekdays and 61.1% (11/18) during weekends. The odds to be informed during the weekend in Phase I were 0.420 times (95% CI 0.194-0.909) the odds to be informed during the weekdays. During Phase II, the odds ratio was 0.711 (95% CI 0.239-2.118) and no longer significantly different from 1 (p = 0.540), which suggests that the TFCP became more embedded in the routine work structure of the ED.

Additional file 2 Table 2. Stratified logistic regression analysis for implementation phase.

| **Phase** | **Variable** | **B** | **S.E.** | **Wald** | **df** | **p-value** | **OR** | **95% CI** |
| --- | --- | --- | --- | --- | --- | --- | --- | --- |
| Phase I | Weekday or weekend | -0.868 | 0.394 | 4.847 | 1 | 0.028 | 0.420 | 0.194 – 0.909 |
|  | Constant | -0.398 | 0.197 | 4.068 | 1 | 0.044 | 0.672 |  |
| Phase II | Weekday or weekend | -0.341 | 0.557 | 0.375 | 1 | 0.540 | 0.711 | 0.239 – 2.118 |
|  | Constant | 0.793 | 0.276 | 8.231 | 1 | 0.004 | 2.211 |  |

B = Regression coefficient, S.E. = Standard Error, df = degrees of freedom, OR = Odds Ratio and 95% CI = 95% Confidence Interval
