# Supplementary figures and images for: Strategies for implementation of a transmural fall-prevention care pathway for older adults with fall-related injuries at the emergency department
Source: BMC Emerg Med. 2024 Oct 11;24:188. doi: 10.1186/s12873-024-01085-9 (PMC11470610; doi:10.1186/s12873-024-01085-9)

# Blueprint Transmural Fall-Prevention

## Design of the care pathway

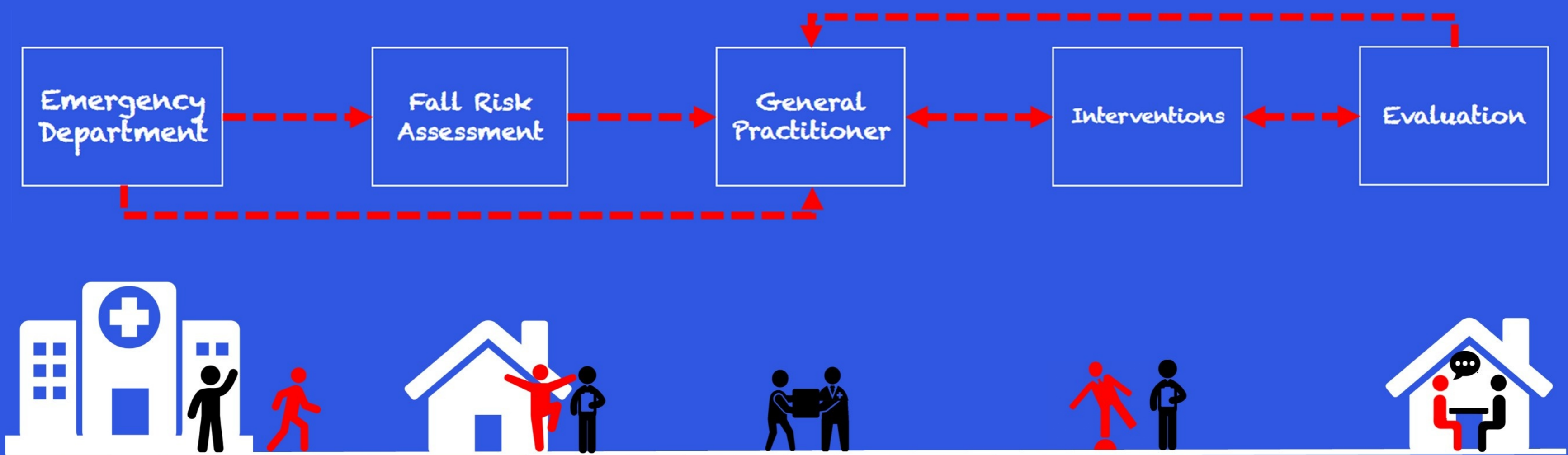

## Implementation process

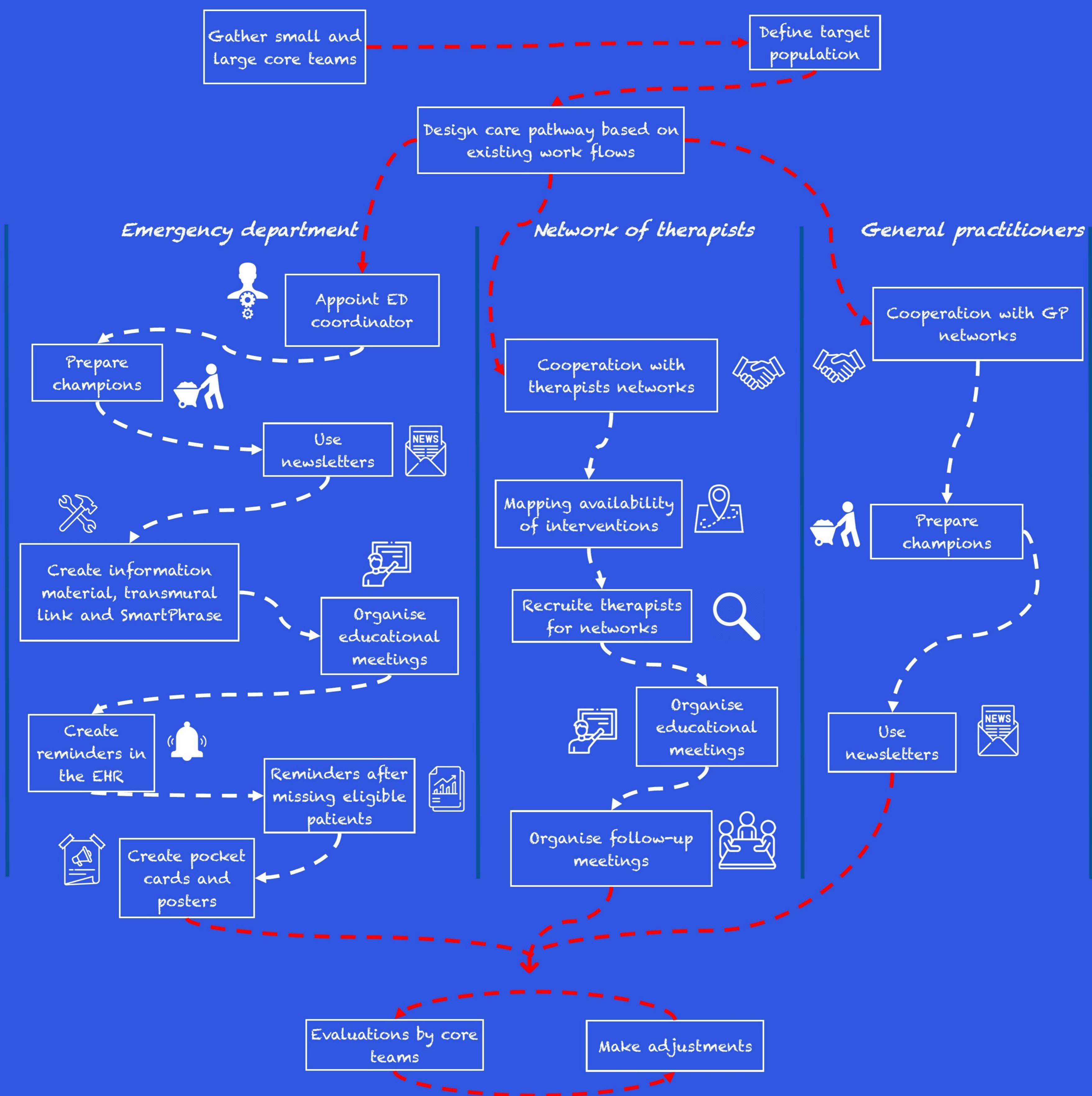

Supplement: Supplementary file 3 — Supplementary Material 3. [file 12873_2024_1085_MOESM3_ESM.pdf]
